# Characteristics of an Unscheduled Emergency Department Revisit Within 72 hours of Discharge

**DOI:** 10.7759/cureus.23975

**Published:** 2022-04-09

**Authors:** Rajesh Sah, LR Murmu, Praveen Aggarwal, Sanjeev Bhoi

**Affiliations:** 1 Emergency Department, BP Koirala Institute of Health Sciences, Dharan, NPL; 2 Emergency Department, All India Institute of Medical Sciences, New Delhi, IND

**Keywords:** discharge, unscheduled, quality, emergency department, revisit

## Abstract

Background

An unscheduled emergency department (ED) revisit is defined as a patient presenting to the ED with the same problem within 72 hours of discharge. The revisits result in overcrowding and compromise the care provided by the ED. We assume that the poor quality of care provided by the ED is the reason for revisiting. However, the circumstances surrounding these revisits are not well-understood. We conducted this study to understand the characteristics associated with the revisits.

Objectives

We aimed to identify the common causes of ED revisits within 72 hours of discharge and determine the outcome of these patients during the revisit.

Methods

We conducted a prospective observational study at a tertiary care center from July 2015 to June 2017, including patients presenting at the ED within 72 hours after their first visit. Our study selected 50 patients using a simple random sampling method and identified the leading causes of revisit as doctor-related, patient-related, and illness-related.

Results

We found that 56% (28/50) of patients returned to the ED for illness-related reasons, 26% (13/50) for doctor-related reasons, and 18% (9/50) for patient-related reasons. In addition, we found that 62% (31/50) of patients who returned to the ED within 72 hours required in-patient admission.

Conclusion

The most common cause of ED revisit was illness-related causes, and more than half of the patients during a revisit required in-patient admission. The modifiable causes of the ED revisit, such as doctor-related and patient-related factors, were discovered in this study. These findings may aid in reducing ED revisits and improving the ED quality.

## Introduction

An unscheduled revisit is defined as a patient who comes to the emergency department (ED) with the same problem within 72 hours of discharge [1-2]. A patient coming to the ED within 72 hours of their first visit may contribute to crowding and indicate a failure to give a proper assessment, treatment, and follow-up instructions [3]. Unplanned returns are considered quality indicators and tools for improving patient care in the ED [4]. It is known that ED revisits contribute to overcrowding, increased wait times, and impaired quality of care for those with urgent needs [5]. When patients return to the ED shortly after being seen, it is usually assumed that their initial assessment or treatment was inadequate [6]. However, there is a lack of understanding of the circumstances of these revisits. Patients who return to the ED within 48-72 hours of their initial visit are described as a population at high risk for errors in diagnosis or physician judgment in their management [7]. Various international studies found that the revisit rate ranged from 3% to 4.9% [8-9]. However, to our knowledge, there are no prospective observational studies in India concerning ED revisits. Therefore, we intended to conduct this study to understand the characteristics associated with these unscheduled ED revisits.

## Materials and methods

Our study is a prospective observational study of patients over 15 years of age who returned to the ED within 72 hours of discharge. We aimed to identify the common causes of ED revisit within 72 hours of discharge and determine the outcome of these patients during the revisit. The inclusion criteria were all patients over 15 years of age who revisited the ED within 72 hours of discharge, and the exclusion criteria were patients with age less than 15 years, patients refusing consent, and patients who did not have previous records of the ED visit. We conducted this study at the ED of a tertiary care center. We selected the patients using a simple random sampling method over two years, regardless of the time of day or month. We selected the patients who had visited the ED within the last 72 hours. We applied the exclusion criteria to recruit the patients. The doctors on duty were required to document the complaints, the patient’s vital signs, investigations, and treatment advised in the patient’s case files, and they were supposed to provide an appropriate discharge paper to the patient and advise the patient about their subsequent management from the outpatient department. We reviewed the case files for this study. A detailed pro forma form was filled, including patient demographic details, triage category, the time and date of arrival and discharge, complaints, examination signs, investigations advised with their reports, diagnosis, management, and patient disposition at each visit. The cause of revisit was then judged and classified as being related to doctor, patient, or illness, which we adapted from the methods of Wu et al. [6]. The cause of revisit was decided by independent observers who were not a part of the research.

The following are definitions of the doctor, patient, and illness-related causes.

Doctor-related causes of revisit

Missed Diagnosis

The diagnosis made at the first and later visits was not the same. It may have happened if we did not conduct appropriate investigations at the first visit or failed to review the reports.

Treatment Error

The doctor made the correct diagnosis during the initial visit but made an error during the treatment, and he did not provide appropriate treatment as indicated.

Patient-related causes of revisit

Compliance

The patient was non-compliant with prescribed medications and advice on discharge.

Left Against Medical Advice (LAMA)

The patient left the ED against medical advice.

Did Not Follow Up in the Outpatient Department (OPD)

The patient did not follow up in the outpatient department despite being advised to do so during the first visit.

Did Not Get Admitted

The patient denied medical advice to get admitted to other hospitals due to the non-availability of a bed.

Illness-related causes of revisit

Complication of Disease

We treated the patient appropriately during the first visit, but the reason for the revisit was a complication of disease despite being treated adequately during the initial ED visit.

Recurrent Disease

The patient was treated adequately during the first visit, but the revisit is associated with recurrent exacerbations of a disease process, for example, asthma, COPD, or seizure disorder.

Progression of Disease

The patient was treated appropriately during the first visit and given proper discharge advice, but the cause of the revisit was that the patient’s condition worsened despite the proper treatment given at the first visit.

If the revisits had illness-related and doctor-related causes, illness-related and patient-related, or patient-related and doctor-related causes simultaneously, we considered it to be due to the latter cause. The outcome of the revisit was evaluated for final dispositions of the patient if the patient was admitted, discharged after being treated in the ED, asked to follow up in the OPD, LAMA, or died. We studied the factor associated with the patient’s revisit in the ED concerning chief complaints, age, sex, co-morbidities, and triage category. The triage at our institute is done according to the All India Institute of Medical Sciences (AIIMS) triage protocol [10]. Statistical analysis to describe the characteristics of the ED revisit was done by recording the data on a predesigned pro forma and managed on a Microsoft Excel sheet (Microsoft Corporation, Redmond, WA). We summarised and analyzed the data using the statistical package STATA software (version 12; College Station, TX: StataCorp LP). We expressed the data as numbers and or percentages as appropriate.

Ethical issues

We took informed consent from the subjects before the study. The subjects had the right to withdraw consent from participation during the study. The subject’s participation and non-participation in the study had no bearing on their treatment. We did not perform any experimental procedure on the subjects and obtained ethical clearance from the ethics committee of the institute.

## Results

Fifty patients were recruited for the study who presented to the ED within 72 hours of discharge. Among the patient revisiting the ED within 72 hours, all 50 patients had a single revisit within 72 hours. The demographic data of all patients under study are presented as follows.

Age distribution

We found the younger age group of patients, between 16 and 45, to contribute 80% of revisits. Table 1 depicts the age distribution of patients during the revisit.

**Table 1:** Age distribution of patients

Age	Frequency	Percentage
16-30	26	52
31-45	14	28
46-60	5	10
61-75	5	10
Total	50	100

Gender distribution

Among the patients recruited for the study, we found male patients (58%) revisit more than female patients (42%).

Triage category

Among patients under study, we analyzed the color-coded triage category based on severity and found the yellow triage category to represent 82% of the revisits. Table 2 depicts the triage category of patients during the first visit.

**Table 2:** Triage category of patients during the first visit

Triage category	Frequency	Percentage
Yellow	41	82
Red	8	16
Green	1	2
Total	50	100

Causes of emergency department revisit

Among patients recruited for the study, we found 56% (28/50) of the patients revisit the ED for the illness-related cause while the doctor-related cause was 26% (13/50) and 18% (9/50) of cases were related to patient factors. Among doctor-related causes, we found 20% (10/50) to revisit because of missed diagnosis, and 6% (3/50) were found to revisit because of treatment error. Among patient-related causes, we found 14% (7/50) cases were the ones who did not get admitted to a referred government hospital while 4% (2/50) were those who left against medical advice during their first visit. Among illness-related causes, we found 48% (24/50) cases revisited because of chronic disease progression and 8% (4/50) revisited because of recurrent disease. Table 3 depicts the distribution of patients based on causes of revisit, and Table 4 depicts the distribution of patients based on the subcategory of a revisit.

**Table 3:** Distribution of patients based on causes of revisit

Causes	Frequency	Percentage
Illness-related	28	56
Patient-related	9	18
Doctor-related	13	26
Total	50	100

**Table 4:** Distribution of subcategory of causes of the revisit

Causes of revisit	Subcategory of causes	Percentage
Doctor-related (26%)	Missed diagnosis	20
	Treatment error	6
Patient-related (18%)	Left against medical advice	4
	Did not get admitted to referred hospital	14
Illness-related (56%)	Recurrent disease	8
	Progression of disease	48

Final disposition of patients during their second visit: Among patients under study, 18 patients (36%) were discharged with advice to visit the outpatient department. Nineteen patients (38%) were transferred to another hospital because of the non-availability of beds, 12 patients (24%) were admitted for further management, and one patient (2%) succumbed to illness during their second visit. Table 5 depicts the final disposition of patients during revisit.

**Table 5:** Final disposition of the patients during the revisit

Final disposition	Frequency	Percentage
Discharged with medicine and advice to visit OPD	18	36
Transferred to another government hospital	19	38
Admitted to our institute	12	24
Succumbed to illness	1	2
Total	50	100

## Discussion

The revisit is a quality indicator of the ED. We studied these revisits and stratified the causes according to the doctor, patient, and illness-related cause, and we studied the final disposition of these patients.

Our research found that 52% of the patients fell into the 16-30 age group, followed by 28% in the 31-45 age group. Interestingly, these results are consistent with the study done by Kuan WS et al., who found that patients aged 21 to 30 represent 29.8% and those aged 16-20 represent 14.6% of the total population [11]. According to our findings, 82% (41/50) of patients received a yellow triage while 16% (8/50) had a red triage, implying that most patients are discharged prematurely due to under-triaging.

In our study, illness-related causes accounted for 56% (28/50) of the revisits. Our findings are consistent with those of Linden et al., who discovered that the primary reason for revisiting was illness (49%) [12]. Our findings are also consistent with those of another study conducted by Imsuwan, who discovered that the most common reason for revisit was illness-related (60.6%), followed by doctor-related causes (28.3%), and patient-related causes (8.5%) [13]. Another study found doctor-related causes accounted for 7.8% while disease-related causes accounted for 79% [14]. Furthermore, our research found that 62% of patients who returned to the ED required in-patient admission. Our findings are consistent with a study on unscheduled returns conducted by S Nunez et al., who found that nearly half of the patients with an unscheduled revisit got admitted to other hospital departments [1]. Our study found that 52% of patients who returned to the ED had some form of co-morbid illness, with 26% having some form of malignancy and 10% having tuberculosis. Our findings are consistent with Loi et al., who discovered that pre-existing co-morbidities are risk factors for revisits [15]. We conclude that illness-related causes account for more than half of the cases, with doctor-related missed diagnosis and treatment errors contributing to revisits. Sri-on et al. discovered that missed diagnosis and treatment errors were associated with half of the cases in their study [9]. Rising et al. discovered that post-discharge factors, such as perceived inability to access timely follow-up care and uncertainty and fear about disease progression, are primary motivators for ED revisits [16]. We also conclude that a high-quality ED discharge system can reduce premature discharge and re-admissions. The only limitation of our research is the small sample size.

## Conclusions

The most common cause of ED revisit was the illness-related cause. More than half of the patients during revisit required in-patient admission. In this study, doctor-related factors and patient-related factors were found as controllable causes of emergency department revisit. To reduce ED revisits and improve the quality of the emergency department, we can act on modifiable causes of a revisit. We suggest that patients receive proper education at discharge, including information regarding the need for a follow-up visit to the appropriate super-specialty department if necessary. We also suggest that patients are well-informed about the diagnosis, treatment, and prognosis. To validate the findings of our study, we believe that more research with a bigger sample size is required.
